# Monthly certifications of vision impairment in England and Wales in 2020 and 2021: comparison with years prior to the COVID-19 pandemic

**DOI:** 10.1038/s41433-022-02300-2

**Published:** 2022-11-05

**Authors:** Omar A. Mahroo, Wen Xing, Snezana Lazarevic, Antra Zekite, Declan Flanagan

**Affiliations:** 1 NIHR Biomedical Research Centre at Moorfields Eye Hospital and the UCL Institute of Ophthalmology, London, UK; 2 Institute of Ophthalmology, University College London, Bath Street, London, UK; 3 Section of Ophthalmology, King’s College London, St Thomas’ Hospital Campus, London, UK

**Keywords:** Epidemiology, Vision disorders

The effect of the pandemic on non-COVID-19-related conditions is a subject of continued investigation [1, 2]. Support services for patients with sight loss have also been affected [3]. Individuals meeting specific criteria are eligible for a Certificate of Visual Impairment (CVI) [4]; certification can facilitate access to support which can substantially and positively impact quality of life. Where patients (parents or guardians in case of children) have given consent, a copy of the certificate is sent to the Royal College of Ophthalmologists Certifications Office based at Moorfields Eye Hospital.

We explored monthly certifications received by the office (for England and Wales) over the 5-year period from Jan 2017 to Dec 2021. Figure 1A plots monthly certifications against calendar month. Monthly certifications were consistently >1800 in the years 2017–2019, but in 2020, certifications fell dramatically in April, May and June (607, 509 and 539 certifications respectively; these represented <30% of the average for the same calendar months in 2017–2019). Monthly certifications were <1800 also in July and August of 2020 (1231 and 1267 certifications respectively) and in January and February of 2021 (1580 and 1579 respectively).

**Fig. 1:**
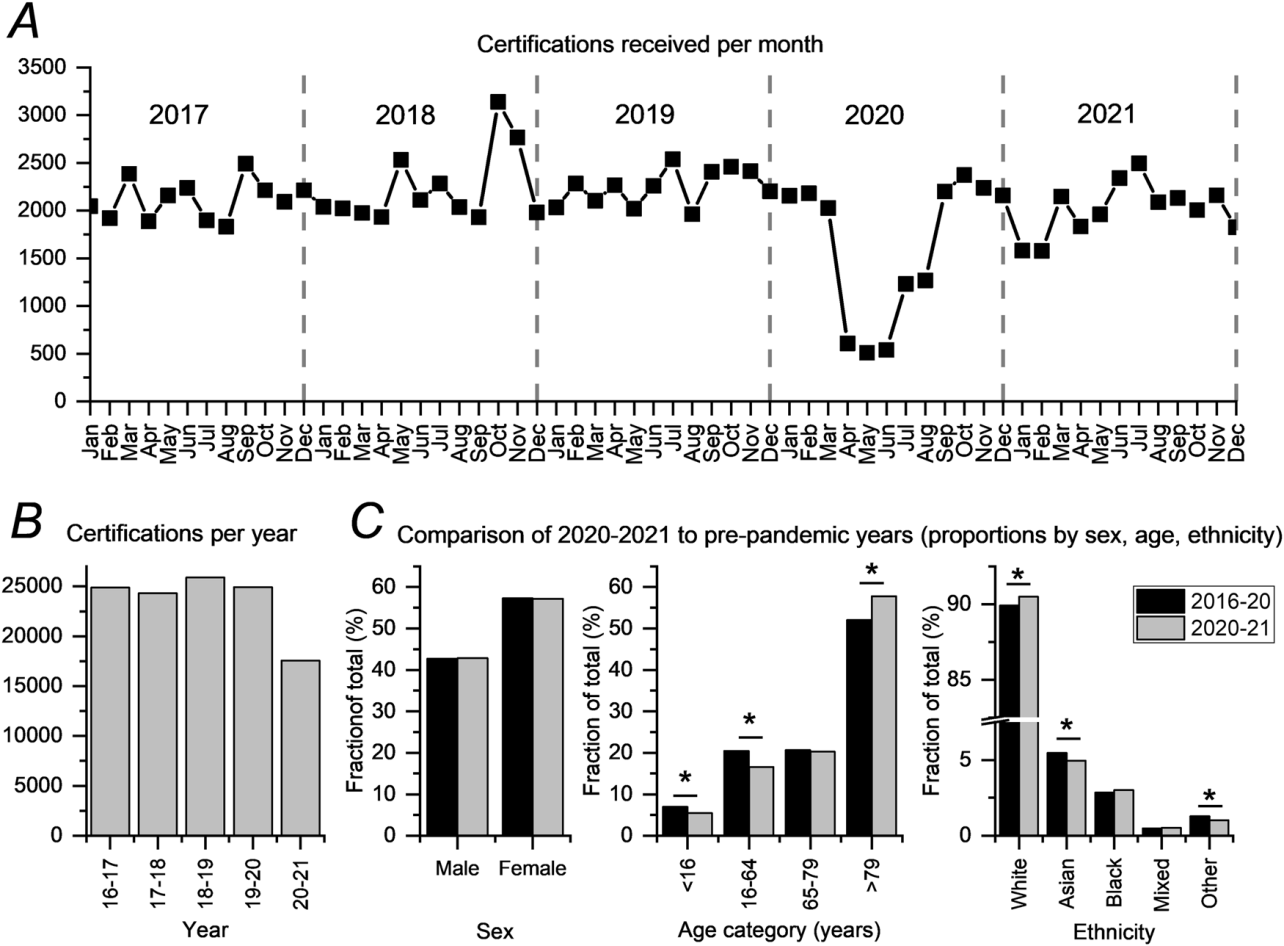
Certifications of visual impairment in England and Wales before and during the COVID-19 pandemic. **A** Numbers of certifications received each month by the Certifications Office between January 2017 and Dec 2021. **B** Annual numbers of certifications between 2016–2017 and 2020–2021 (here, each year starts from 1 April). **C** Proportions of certifications (by sex, age group and ethnicity) for 2020–2021 compared with the preceding 4 years. In each case, the fraction is of the total after excluding certifications for which the relevant demographic data are unknown. For age and sex, these were unknown in <1% of certifications. Ethnicity was unknown in 13.5% of certifications. *Denotes nominal significance at *p* < 0.05. For the upper panel (**A**), dates refer to date of receipt by the Certifications Office. For the lower panels (**B**, **C**), dates relate to recorded date of certification.

We then sought to explore trends by sex, age and ethnicity. These data were available for certifications grouped by year (1 April to 31 March of the following year) from 2016–17 to 2020–21. Figure 1B shows total certifications by year. April 2020 (the first month in which certifications dramatically fell) was the first month of the 2020–21 year, and so this year was compared to the 4 previous years. Figure 1C illustrates these comparisons: the sex distribution was unchanged; those aged ≥80 accounted for a higher proportion of CVIs in 2020–2021 (*p* < 0.0001), whilst children and working age adults accounted for lower proportions, compared with pre-pandemic years (*p* < 0.0001). Whilst the overall ethnicity distribution appeared similar, the white proportion was higher in 2020–2021 (*p* = 0.03), whilst Asian and “Other” proportions were lower, compared with the preceding 4 years (*p* = 0.01 and *p* = 0.006 respectively).

In conclusion, we observed a marked (>70%) reduction in certification forms received in April, May and June of 2020, coinciding with the first national lockdown. As certifications can enable access to support, this is another manifestation of the consequences of the pandemic for patients with vision loss. A similar magnitude reduction was not observed for subsequent lockdowns. A study in Northern Ireland also found a reduction in certifications in March to June 2020 compared with the same period in 2019 [5]. In our further exploration, we did not find a disproportionate impact by sex. There was no evidence of a disproportionate barrier to certification affecting the elderly (those over 80 in fact made up a larger fraction of certifications compared with pre-pandemic years). There were falls observed in the fraction of certifications comprising those of Asian or “Other” ethnicity; this might represent differential barriers to access, but other factors could contribute. Limitations of this study include certifications where demographic data were unknown: for age and sex, these constituted less than 1% of certifications, whereas ethnicity was unknown in 13.5%.

## Data Availability

Data relating to certification of visual impairment are publicly available at https://fingertips.phe.org.uk/profile/public-health-outcomes-framework. The data processed for the analysis in this manuscript are available on request.
